# General Agreement

**Published:** 1921-05-28

**Authors:** 


### THE HOSPITAL PAGEANT MEETING.

### General Agreement.

The meeting of the Hospital Secretaries in the London area, held at the offices of The Hospital on Friday, May 20, to consider the question whether a Hospital Pageant is practicable was attended by representatives of the following hospitals: St. Bartholomew's, Middlesex, King's College, St. George's, Westminster, St. Mary's, Seamen's, Prince of Wales's, General, Metropolitan, Hampstead General, Great Northern Central Hospital for Sick Children, East London for Children, Royal Eye, King Edward Memorial, Ealing, Samaritan Free, Royal Dental, Kensington Dispensary and Children's, Jewish Maternity, Western Skin, Kensington and Fulham, City of London Maternity, Miller General, Belgrave, London Fever, East Ham, St. John's for Skin, National for Diseases of the Heart, St. Andrew's, Roll of Honour, Florence Nightingale, Jewish Maternity Home and Sick Room Helps Society, Nelson, London Homœopathic, Central London Ophthalmic, Grosvenor for Women, London Skin, Victoria for Children, Queen Mary's for East End, All Saints' London Lock, Finchley Cottage, East End Mothers', Elizabeth Garrett Anderson, British for Mothers and Babies, and South London.

Mr. Sidney M. Quennell (Westminster Hospital) having been unanimously elected to the chair, Mr. Gilbert Panter (Great Northern Central Hospital) explained the object of the meeting, which arose out of the article in The Hospital of May 7. Diffidently referring to his own part in convening the meeting, he laid stress on the point that to keep the voluntary hospitals going the only way is by combined effort at certain times in the year. The object of the meeting was to see whether the proposal of a Hospital Pageant is practicable, and, if so, to draw up a definite scheme for it. He has received a large number of letters from the secretaries of various London hospitals, all of whom agree with the proposal, though some think it rather short notice for this year, and all emphasise the fact that whatever is done the Pageant should be of a dignified nature, and not a so-called demonstration. Mr. Bone (Miller General Hospital) detailed the demonstrations already arranged for the period from June to October on behalf of various hospitals by the South-Eastern Hospitals Demonstration Committee, and Monsignor Carton de Wiart (St. Andrew's Hospital) suggested that November 13 might be a good date. Mr. James McKay (Hospital for Sick Children, Great Ormond Street) emphasised that the machinery for and details of the organisation should be left to whomever is appointed to deal with the matter; he believes that something of the kind suggested, properly worked, will appeal to the majority of Londoners, and be of material benefit to the hospitals, and hopes that the experience of a master-mind may be made available.

After one or two questions, Mr. R. J. Coles (King's College Hospital) proposed "That it is desirable to hold a Hospital Week, including a Pageant." Mr. Scrivener (Queen Mary's Hospital) asked for a definition of London hospitals. Mr. Panter suggested hospitals within the Metropolitan area covered by the King's Fund, and, in reply to Mr. Van Someren (King Edward Hospital, Ealing), that it should include all hospitals, whether large or small, within the King's Fund area. Mr. Coles' resolution was seconded and carried *nem. con.* Mr. Bone then suggested that any decision of the meeting should be sent to the three Funds—King Edward's, the Hospital Sunday, and the Hospital Saturday Funds—in order to get combined action and co-operation. On a question as to expenses, Mr. Panter suggested a subscription by all the hospitals at a certain rate per bed. Mr. Scrivener and Mr. Wilcox (East London Hospital for Children) respectively moved and seconded a resolution that the decision of the meeting should be sent to the Committees of the London hospitals asking for its consideration. This was supported by Col. Parkes (St. Mary's Hospital), who suggested that in addition to the organisation of a Pageant the co-operation of the large stores should be secured in certain directions. Capt. Whitney (National Heart Hospital) desired to include in the resolution some indication of the proposed method of finance, so that the Hospital Boards may have all the facts before them. Mr. Panter suggested that the Regional Committee of the British Hospitals Association might be asked to appoint the Pageant Committee, and Mr. Bone that this point should wait upon the issue of Lord Cave's Report in order to see who it proposes should act as the central hospital body for London. Mr. Panter remarked that whatever Lord Cave's Committee may report there will still be necessity for propaganda. In reply to Monsignor Carton de Wiart's reference to a Red Cross appeal in October, Mr. Panter urged that as it is probable the British Red Cross will devote the whole of their proceeds towards helping provincial hospitals, and as London hospitals badly need any money collected here, the place for the Red Cross effort is the provinces. He does not, however, think it necessarily follows that a great deal of profit will be made out of the Pageant; its value is in what follows. It is the advertisement that is wanted. After the question of the participation of individual hospitals in profits had been raised and other points, it was moved by Mr. Wilcox, and seconded by Mr. Churchfield (St. George's Hospital) that before sending the resolution to the Hospital Boards it should be sent to the three Funds for their consideration. This was carried unanimously. When the replies are received from the Funds another meeting of hospital representatives will be called. Meanwhile, Mr. Panter suggested that Mr. H. E. Smithers, General Manager of The Hospital, should be appointed to receive correspondence arising out of the present meeting. This was agreed to, and after votes of thanks to the Chairman for presiding, to Mr. Panter and Mr. Smithers for their trouble in calling the meeting, and to The Hospital for the use of the room, the proceedings terminated.

